# Recent Overview of Resveratrol’s Beneficial Effects and Its Nano-Delivery Systems

**DOI:** 10.3390/molecules27165154

**Published:** 2022-08-12

**Authors:** Raghvendra A. Bohara, Nazish Tabassum, Mohan P. Singh, Giuseppe Gigli, Andrea Ragusa, Stefano Leporatti

**Affiliations:** 1 CÚRAM, SFI Research Centre For Medical Devices, National University of Ireland Galway, H91 W2TY Galway, Ireland; 2 Centre for Interdisciplinary Research, D.Y. Patil Education Society, Deemed to be University, Kolhapur 416006, India; 3 Centre of Biotechnology, University of Allahabad, Prayagraj 211002, India; 4 CNR NANOTEC—Istituto di Nanotecnologia, 73100 Lecce, Italy; 5 Department of Mathematics and Physics, University of Salento, 73100 Lecce, Italy; 6 Department of Biological and Environmental Sciences and Technologies, University of Salento, 73100 Lecce, Italy

**Keywords:** resveratrol, polyphenols, drug delivery, nanocarriers, nanomedicine

## Abstract

**Highlights:**

1. Resveratrol is an antioxidant and exhibits numerous potential therapeutic applications. However, it suffers from low water-solubility, degradation, and poor bioavailability. The conventional dosage form of resveratrol shows various limitations, such as prolonged therapy, erratic bioavailability, and absence of effective drug concentrations in tissues.
2. Nanocarrier-based delivery systems are being studied extensively to target tissues and cells to improve the therapeutic potential of poorly soluble molecules by enhancing their bioavaila-bility, solubility, and retention time.
3. Resveratrol can act as a potential anti-cancer agent and lowers the progression of cancer disease.

**Abstract:**

Natural polyphenols have a wide variety of biological activities and are taken into account as healthcare materials. Resveratrol is one such natural polyphenol, belonging to a group known as stilbenoids (STBs). Resveratrol (3,5,4′-trihydroxy-*trans*-stilbene) is mainly found in grapes, wine, nuts, and berries. A wide range of biological activities has been demonstrated by resveratrol, including antimicrobial, antioxidant, antiviral, antifungal, and antiaging effects, and many more are still under research. However, as with many other plant-based polyphenol products, resveratrol suffers from low bioavailability once administered in vivo due to its susceptibility to rapid enzyme degradation by the body’s innate immune system before it can exercise its therapeutic influence. Therefore, it is of the utmost importance to ensure the best use of resveratrol by creating a proper resveratrol delivery system. Nanomedicine and nanodelivery systems utilize nanoscale materials as diagnostic tools or to deliver therapeutic agents in a controlled manner to specifically targeted locations. After a brief introduction about polyphenols, this review overviews the physicochemical characteristics of resveratrol, its beneficial effects, and recent advances on novel nanotechnological approaches for its delivery according to the type of nanocarrier utilized. Furthermore, the article summarizes the different potential applications of resveratrol as, for example, a therapeutic and disease-preventing anticancer and antiviral agent.

## 1. Introduction

Plant-based natural products are commonly used as an alternative to modern medicines. Since the beginning of human life, herbal medicines have developed and continued to exist until today. Awareness of this herbal medicine is considered to be the foundation of modern medicine [1]. The use of state-of-the-art technologies, such as purification techniques and computer bioinformatics tools, has helped to classify the plant’s active ingredients and the molecular pathways that are potentially affected [2]. Natural materials display impressive qualities, such as excellent chemical diversity, biological and chemical activities with a macromolecular precision, and absent or low toxicity. These properties make them ideal candidates for developing novel drugs [3]. Additionally, theoretical studies have helped to envisage drug–target interactions and to build new drug generations, such as in the targeted drug discovery and drug delivery [4]. Despite the enormous potential of phyto-components, the pharmaceutical companies are not so active in investing and researching with this approach. One possible reason is the lack of an in-depth molecular mechanism understanding and of viable carriers that ensure onsite delivery. These concerns have been extensively addressed in the recent years. Detailed molecular work has been carried out to study the in-depth action of plant-based natural materials [3,4]. However, concerns associated with the toxicity of some metabolites and their interaction with the desired target are still a problem, thus requiring additional studies to find viable alternatives. Hence, many natural compounds are not clearing the preclinical and Phase I clinical trials [5,6,7]. The possible solution to this problem can be the development of a nanocarrier system which can carry natural compounds and ensure their onsite delivery without causing toxicity [8]. Recently, nanotechnology has demonstrated these capabilities and it is considered to be the future of drug delivery systems. One successful example is the development of liposomal doxorubicin (Doxil^R^), which has shown massive success in treating metastatic breast cancer [9].

Different carriers, micelles, liposomes, and nanoparticles (NPs) have been developed as natural product delivery systems, and they have shown promising results [10]. However, concerns related to polyphenols’ nanoencapsulation have been reported due to the varying structures, solubility, and fast oxidation under physiological conditions [11]. Therefore, it is crucial to consider these impairing alterations on the polyphenol molecules when designing the nanocarrier.

**Figure 1:**
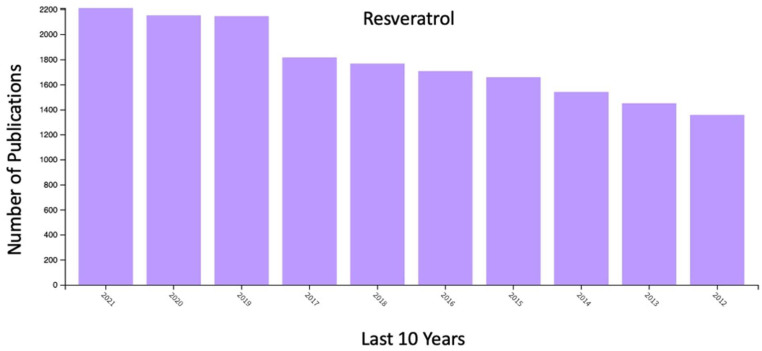
Cumulative research articles related to resveratrol grouped by year (2012–2020), as determined by the Web of Science^®^ database (last accessed on 30 June 2022).

Among the different natural polyphenols, resveratrol (RES) has shown immense potential due to excellent antioxidant, anticancer, antihypertensive, anti-inflammatory, and antiplatelet aggregation cardioprotective activities [12,13,14,15]. Because of these interesting properties, there is a sudden interest in exploring the full potential of this molecule (Figure 1). 

**Figure 2:**
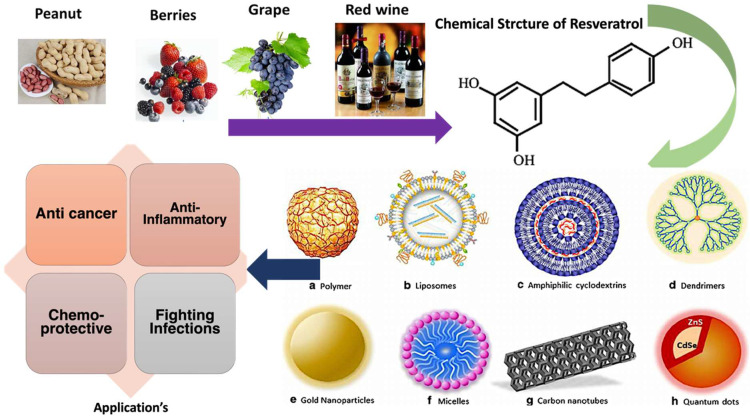
Scheme showing the main sources of *trans*-resveratrol, its chemical structure, the types of nanoformulations used to deliver it, and its beneficial health effects and applications.

This review article, after a brief introduction about natural polyphenols, focuses on the chemistry of RES and nanotechnology’s role in the development of RES delivery systems. The use of RES as anticancer drug is also discussed to understand how the carrier system can play a vital role in exploring the potential of natural phytochemicals, such as RES (see Figure 2).

## 2. Natural Polyphenols

Polyphenols are a phytochemical group with chemical properties and different structures ranging from simple molecules to polymers with high molecular weight [16]. Polyphenols are secondary plant metabolites, and their primary role is to attract pollinators and protect plants from insects, ultraviolet radiation, and microbial infection [17]. Polyphenols comprise a phenolic system with at least one phenyl ring and one or more hydroxyl substituents. Polyphenols are present in vegetables, fruits, herbs, spices, beverages such as tea and wine, chocolates, and whole grains [18].

### Classification of Polyphenols

Generally, polyphenols are mainly categorized into flavonoids and nonflavonoids, but according to the aglycone chemical structures, they can be also classified as phenolic acids, lignans, and stilbenes [16].

**Flavonoids.** It is the largest group of phenolic compounds and mostly present in fruits. The structures consist of two aromatic rings bound with a three-carbon bridge to form an oxygenated heterocycle. The biological activities, such as anticancer, anti-inflammatory, and antioxidant ones, depend on the flavonoids’ structural difference and glycosylation pattern [19]. Based on the number, position of the –OH groups and degree of oxidation of the central ring, flavonoids can be further divided into subclasses of flavones, flavonols, flavanones, flavanols, isoflavones, and anthocyanidin. Flavones, such as apigenin and luteolin, are found in celery, parsley, and some herbs. The polymethoxylated flavones (e.g., tangeretin and nobiletin) are found only in the tissues and peels of citrus fruit, such as oranges, grapefruit, and tangerines. The flavones’ characteristic feature is to have methylated –OH groups, which increases their metabolic stability and enhances their oral bioavailability [20]. Flavonols are one of the most ubiquitous flavonoids found in capers, saffron, dried Mexican oregano, yellow and red onions, and spinach.

Two representatives of this subclass are quercetin and kaempferol. Naringenin and hesperetin are valuable flavanones found in food. The maximum concentrations of flavanones are present in citrus fruits and dried herbs, and they are responsible for the bitter taste of fruits [21]. Isoflavones are present in legumes, and the chief source is soybeans, having substantial amounts of daidzein and genistein. Isoflavones show pseudo-hormonal characteristics because of their structural similarity to oestrogen, and their supplements can bind to the oestrogenic receptors and, as such, be used as potential substitutes for conventional hormone therapy [22]. Among all flavonoids, flavanols are the most complex subclass, being able to form monomers, oligomers, polymers, and other derivative compounds. They are responsible for the bitterness of chocolate and the acid-based character of some fruits and beverages. Anthocyanidins are a subgroup of flavonoids responsible for the colour of flowers, leaves, fruits, and roots. Pelargonidin, delphinidin, peonidin, petunidin, malvidin, and cyanidin are common anthocyanidins distributed in fruits and vegetables [23].

**Phenolic acids.** They can be divided into two groups, led by benzoic and cinnamic acid. Gallic and ellagic acid are the essential derivatives of benzoic acid, and they are found in cranberries, raspberries, pomegranates, and nuts. On the other hand, cinnamic acids’ essential derivatives are ferulic, coumaric, caffeic, and sinapic acid [24].

**Lignans.** The highest amount of lignans is found in flaxseeds, grains, and certain vegetables. In plants, they are typically found as glycosides and are converted by intestinal bacteria to give metabolites having oestrogenic activity [25].

**Stilbenes.** They are phytoalexins produced by plants in response to injury and infections. In the human diet, they are present in low quantities, and only resveratrol is essential for the human health. Grapes and red wine are an important dietary source of resveratrol. Resveratrol is directly connected to the French paradox. It was observed that French people consume high amounts of saturated fatty acids but they hardly suffer from cardiovascular diseases, having a lower death rate than other European countries. It is thus supposed that the daily consumption of red wine plays a significant role in preventing heart diseases [26].

## 3. Chemistry of Resveratrol

**Figure 3:**
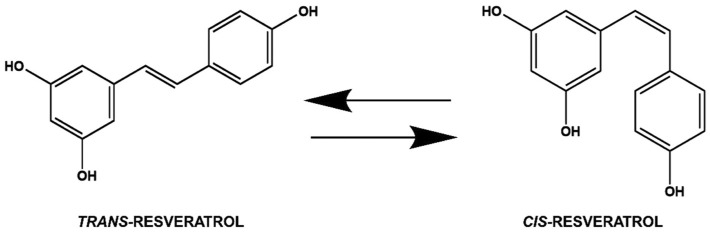
Chemical structures of *trans*- and *cis*-resveratrol.

Resveratrol is a stilbenoid polyphenol with two phenol rings linked to each other by an ethylene bridge. The IUPAC name of resveratrol is E-5-(4-hydroxystyryl)benzene-1,3-diol, but both geometric isomers of resveratrol, the cis and the trans, can be found (Figure 3). 

After exposure to the UV radiation, the *trans* isomer can transform into the *cis* one. However, the *trans* form is dominant because of its occurrence, and it is also more interesting because of its many biological activities. The *trans* form induces cell differentiation, cell cycle arrest, apoptosis, and increased antiproliferation in cancerous cells [27]. The stability of *trans*-resveratrol powder depends on humidity (75%) and temperature (40 °C) in the presence of air. The isomerization of *trans*-resveratrol is influenced by irradiation time, wavelength, temperature, pH, and physical status of the molecule. Waterhouse and co-workers investigated the reaction kinetics [28]. *trans*-Resveratrol’s stability is 42 h in neutral aqueous buffer and 28 days in acidic media when protected from light. At pH 10.0 the initial half-life for *trans*-resveratrol is nearly 1.6 h. The bioavailability of resveratrol is very low. Therefore, modification of its structure has received great attention from the scientific community and various resveratrol derivatives have been synthesized. Nanoformulations containing resveratrol have been considered as a promising approach for retaining its biological functions until delivery [28]. To enhance the oral bioavailability, two delivery systems of resveratrol, i.e., stable lipid nanoparticles and nanostructured lipid carriers, have been developed [29]. Moreover, as compared with the free form, resveratrol nanoparticles improve its solubility and increase its antioxidant potential [30].

In 1940, resveratrol was first isolated from white hellebore (*Veratrum grandiflorum* O. Loes) roots. After that, in 1963, it was isolated from *Polygonum cuspidatum* roots, which are used as antiplatelet and anti-inflammatory agents in traditional Chinese and Japanese medicine [31]. Resveratrol acts as a phytoalexin in plants synthesized in response to UV irradiation, mechanical injury, and fungal attacks. In industry, resveratrol is generally prepared by chemical or biotechnological synthesis from yeasts of *Saccharomyces cerevisiae* [32]. Ninety-two new resveratrol derivatives have been reported from the *Leguminosae*, *Paeoniaceae*, *Dipterocarpaceae*, *Vitaceae*, *Gnetaceae*, *Cyperaceae*, *Polygonaceae Gramineae*, and *Poaceae* families. Among these families, *Dipterocarpaceae* alone contains more than 50 resveratrol derivatives, and it is from seven genera [33]. Because of the broad range of pharmacological effects, resveratrol is sold on the market as a nutritional supplement [34].

## 4. Designing of Nanocarriers for Resveratrol Delivery

Nanocarriers are drug carrier systems usually having <500 nm particle size [35]. They have high surface-area-to-volume ratios, the ability to alter bioactivity of drugs, enhanced pharmacokinetics and biodistribution, and potential site-specific delivery, due to which they have already shown promising results as therapeutic agents in the drug delivery area [36]. In anticancer therapy, several problems arise when chemotherapeutics are delivered through conventional administration routes, such as low specificity, high toxicity, and induction of drug resistance, thus reducing the therapeutic value of most anticancer drugs [37]. To overcome this obstacle, nanocarrier-based platforms have been used to deliver anticancer drugs into the tumours and disturb the tumour microenvironment’s pathophysiology, thus improving the therapeutic efficiency [38].

**Types of nanocarriers.** Nanocarriers can be categorized into three different categories: organic, inorganic, and hybrid nanocarriers. Organic nanocarriers comprise solid-lipid nanocarriers (SLNs), liposomes, dendrimers, polymeric nanoparticles (PNPs), virus-based nanoparticles (VNPs), and polymeric micelles (PMs), whereas inorganic polymers include mesoporous silica nanoparticles (MSNs) and carbon nanotubes (CNTs) (Figure 2).

**Organic nanocarriers.** SLNs increase controlled drug delivery, lack biotoxicity, present high drug-carrying efficiency and good stability, improve the bioavailability of poorly water-soluble drugs, and can be easily produced large scale [39]. SLN nanocarriers have been exploited to deliver anticancer drugs, such as methotrexate, docetaxel, paclitaxel, 5-fluorouracil (5-FU), and doxorubicin [40,41]. Liposome nanocarriers have shown several advantages over conventional therapy. They can increase delivery of the drug, prevent early degradation of the encapsulated drug, improve the performance of the drugs, are cost-effective formulations, and represent an effective treatment with reduced toxicity. They are generally used as carriers for drugs, vaccines, cosmetics, and nutraceuticals [42]. Dendrimers are spherically-shaped, monodispersed macromolecules with an average diameter of 1.5–14.5 nm and unique characteristics [43]. Lai et al. used dendrimers to enhance the efficacy of doxorubicin [44]. They used photochemical internalization technology to enhance the cytotoxicity to cancerous tissue. Polymeric nanoparticles (PNPs) are solid biodegradable polymers, nanosized colloidal particles ranging from 10 to 1000 nm [45]. Based on the structure, PNPs are categorized as nanospheres and nanocapsules. Nanospheres are a type of PNPs entrap the drug in the polymer matrix whereas, in nanocapsules, the drug is dispersed in the liquid core of oil or water encapsulated by a solid polymeric membrane [46]. PNPs are highly versatile. Examples of PNPs as a carrier for anticancer molecules are PLGA, PCL, chitosan, PLA, and alginate NPs [47,48]. Polymeric micelles (PMs) are 10–100 nm nanosized colloidal particles formed by the self-assembly of synthetic amphiphilic copolymers in an aqueous solution [49]. They have a core–shell structure with a hydrophobic core and hydrophilic shell [50]. The hydrophobic core of PMs captures the hydrophobic drugs and controls the drug release, thus increasing their water-solubility, whereas the hydrophilic shell of the PMs stabilizes the core and controls the in vivo pharmacokinetics [51]. Various chemotherapeutic agents, such as methotrexate, paclitaxel, docetaxel, cisplatin, and doxorubicin, have successfully been formulated in PMs for cancer treatment. Poloxamer-based micelles, PEGylated polyglutamic acid micelles, PEG-PLA micelles, and PEG-PAA micelles have been used as delivery systems [52]. VNPs are self-assembled protein cages with a diameter <100 nm, having uniform nanostructures. Recently, VNPs have been widely explored for various applications, such as drug delivery, vaccination, imaging, gene therapy, and targeting [53].

**Inorganic nanocarriers**. From the last few years, silica materials hold great promises in nanomedicine due to their straightforward and cost-effective synthesis and functionalization, and their multiple uses. Among silica materials, mesoporous silicas are catching the attention of researchers in drug delivery because of their honeycomb-like structure with hundreds of pores ranging from 2 to 50 nm, with narrow pore size and versatility of loading large amounts of drugs [54]. MSNs possess promise as nanoscale drug carriers because of good biocompatibility, controllable pore diameters, high specific surface area and pore volume, high loading capacity, and good chemical stability, which enhances therapeutic efficacy and decreases the toxicity of drugs [47]. Paclitaxel, camptothecin, doxorubicin, and methotrexate anticancer drugs have been effectively delivered via MSNs [55]. CNTs have a nanoneedle shape, hollow monolithic structure, high mechanical strength, electrical and thermal conductivities, and the ability of easy surface modification [56]. The needle-like shape of CNTs crosses the cell membrane and enters into the cell. One of the drawbacks of CNTs is insufficient water solubility, as well as their toxicity. However, after surface-functionalization, CNTs become water-soluble, biocompatible, less toxic, serum-stable, and ideal nanocarriers for cancer therapy [57]. Examples of the application of CNTs in anticancer drug delivery are methotrexate, paclitaxel, cisplatin, carboplatin, doxorubicin, and mitomycin C [58].

**Hybrid nanocarriers.** Hybrid nanocarriers have been developed to combine the properties of organic and inorganic materials. Surface coating with polyethyleneimine (PEI) increased the cellular uptake of MSNs for efficient nucleic acid delivery [59]. These hybrid nanocarriers prevented premature release of the loaded drug, multidrug resistance, extended the retention of hydrophilic drug cargo, and yielded stimuli-responsive drug release. Desai and co-workers reported the intracellular delivery of zoledronic acid in breast cancer cells [60]. Han et al. developed doxorubicin-loaded, hybrid, lipid-capped MSNs with pH and redox-responsive release of the drug cargo. These hybrid nanocarriers increased the uptake of doxorubicin, thus improving its accumulation, efficiency, and cytotoxicity [61].

The solubility of resveratrol has been shown to be very low, leading to low bioavailability [15] Hence, while designing delivery system for resveratrol, efforts should be made to enhance its bioavailability. This aim has been attempted by using cyclodextrin complexes, dendrimers, liposomes, self-nano-emulsifying drug delivery systems, solid lipid nanoparticles, nanosuspensions, nanocapsules, microparticles and both nano- and micro-encapsulation (Table 1).

## 5. Prophylactic and Therapeutic Applications of Resveratrol

**Figure 4:**
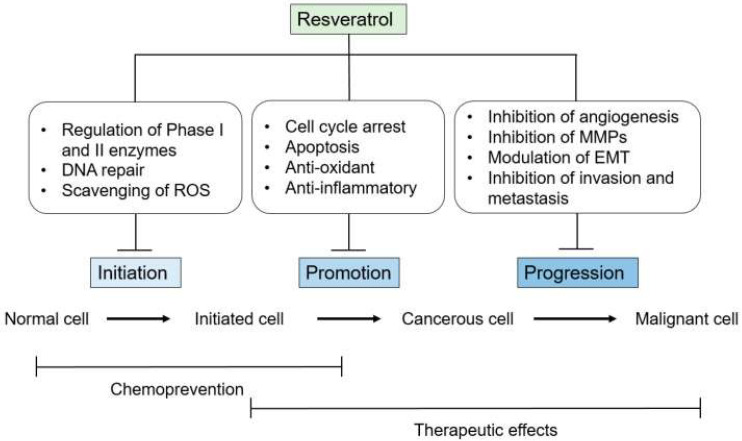
Schematic diagram summarizing the potential mechanisms underlying resveratrol’s anticancer effects (adapted from [15]).

Cancer remains one of the main reasons for mortality in the world. Despite cutting-edge research, the mortality rate has not improved in the last 5 years. More than 1.6 million new cancer diagnoses and approximately 600,000 cancer-related deaths are expected in the United States of America in 2016 alone [67]. Despite the use of novel approaches, such as monoclonal antibodies (MABs) and personalized chemotherapy, the clinical outcomes are not satisfying [68,69]. The complexity of the diseases may justify the failure of cancer treatments. Carcinogenesis is a complicated and multistep process which involves molecular and cellular alterations, which are distinct but closely connected phases of initiation, promotion, and progression [70,71,72] (see Figure 4).

A possible reason for the failure of current cancer therapies, including chemotherapy, radiation, surgery, and immunosuppression, is emergency resistance [73]. Natural molecules are devoid of developing resistance, and delivering therapeutic effects without side effects remains a primary objective in the fight against cancer.

### 5.1. Resveratrol in Cancer Prevention and Treatment

Recent research has suggested that resveratrol can prevent the events linked to the initiation of tumours, which plays a vital role in developing resistance towards the current line of therapeutics. This characteristic of resveratrol has attracted great attention toward this molecule compared to other natural polyphenols. Another important mode of action is the prevention of free radical formation induced by 12-*O*-tetradecanoylphorbol-13-acetate (TPA). It has been seen over the course that TPA is one of the primary carcinogens mostly seen in the liquid tumours (e.g., in human leukaemia HL-60 cells) [74]. The various antioxidant properties of resveratrol have already been described previously [75,76]. Resveratrol is an excellent scavenger of hydroxyls and superoxides as well as the radicals induced by metals/enzymes and generated by cells [77]. It also protects against lipid peroxidation within cell membranes and damages DNA resulting from reactive oxygen species (ROS) [78]. Furthermore, resveratrol can function as an antimutagen, as shown by the inhibition of the mutagenicity of *N*-methyl-*N*′-nitro-*N*-nitrosoguanidine in the *Salmonella typhimurium* strain TA100 [79]. It has been proposed that resveratrol can be a possible chemo preventive agent, and its antimutagenic and anticarcinogenic properties have been demonstrated in several models [80]. Moreover, resveratrol showed a protective role in preclinical models of osteoarthritis and rheumatoid arthritis by reducing the production of pro-inflammatory and pro-degradative soluble factors, and through modulation of cellular and humoural responses [81].

**Figure 5:**
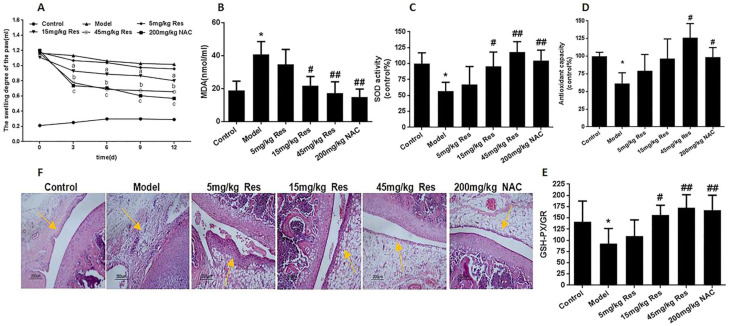
Resveratrol reduced oxidative injury in AA rats after injecting FCA 20 days, treated with 5 mg/kg, 15 mg/kg, 45 mg/kg resveratrol and 200 mg/kg *N*-acetyl-L-cysteine (NAC) for 12 days by continuous intragastric administration. (**A**) Swelling degree of the paw in SD rats after intragastric administration. (**B**) Lipoperoxide levels in the serum from six groups rats. (**C**) SOD activity in serum from six groups of rats. (**D**) Antioxidant capacity in serum from six groups of rats. (**E**) The ratio of glutathione peroxidase and glutathione reductase in serum from six groups of rats. (**F**) HE staining of knee joint in AA rats after administration resveratrol. Values are the means ± SD of at least three independent experiments. * *p* < 0.05 versus control; ^#^ *p* < 0.05, ^##^ *p* < 0.01 versus model. (Reproduced with permission from [82]).

Furthermore, resveratrol could enhance oxidative stress in adjuvant-arthritis (AA) rats and increase mtROS production by reducing the autophagy protein Beclin1, LC3A/B, and oxidative stress protein MnSOD, favouring the apoptosis of FLSs [82] (see Figure 5 for details). 

Resveratrol reduced the expression of heme oxygenase-1 (HO-1) and nuclear factor-E2-related factor 2 (Nrf2), and increased the expression of matrix metalloproteinase (MMP)-2, MMP-9, cyclooxygenase-2, and Toll-like receptor-4 in rats and further inhibited the formation of osteoclasts, the production of inflammation-related proteins, and of circulating ROS in periodontitis rats [83]. Treatment of monosodium iodoacetate (MIA)-injected rats with resveratrol (5 or 10 mg per kg BW) considerably reduced hyperalgesia and reduced the vertical and horizontal movements, additionally reducing any increase in the COX-2 and iNOS mRNA signalling pathways [84]. Very recently, it was also reported that in human bone mesenchymal stem cells, resveratrol suppressed interleukin-1β and thus downregulated metalloproteinase-13 mRNA expression and that it upregulated the chondrocyte markers (aggrecan, Col2, and Sox9 mRNA expression) [85].

### 5.2. Resveratrol in Wound Healing

**Figure 6:**
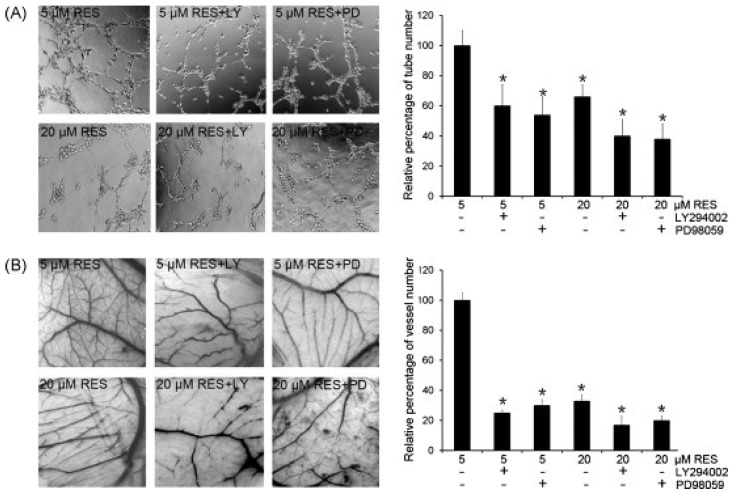
RES-induced angiogenesis was blocked by a PI3K inhibitor and an MEK inhibitor. (**A**) HUVECs were seeded on growth factor-reduced Matrigel-coated plate. Cells were treated with RES (5 μM or 20 μM) in the absence or presence of LY294002 (LY) or PD98059 (PD). The number of tube-like structures was scored. * *p* < 0.05 when compared to 5 μM RES treatment. (**B**) RES-induced angiogenesis was blocked by the PI3K inhibitor and MEK inhibitor in vivo by CAM assay. Cells were treated with RES (5 μM or 20 μM) in the absence or presence of LY294002 (LY) or PD98059 (PD). The number of branching vessels was scored. Each experiment was repeated three times. * *p* < 0.05 when compared to 5 μM RES treatment. (Reproduced with permission from [88]).

Inflammation, proliferation, maturation, and remodelling are the four phases related to tissue integrity process after injury. The proliferation phase, starting within few days after injury, presents healing processes such as angiogenesis, granulation tissue formation, collagen deposition, and epithelialization [86]. Resveratrol seems to be a promising treatment approach, interacting with the healing cascade at different levels [87,88,89]. Several studies suggest that resveratrol could increase wound healing [87,90,91]. Figure 6 shows RES-induced angiogenesis blocked by PI3K and MEK inhibitors in HUVEC cells, as investigated by Wang et al. [88].

Resveratrol-induced vascular endothelial growth factor (VEGF) expression can regulate tissue regeneration and revascularization in wounds. Furthermore, it inhibits the expression of tumor necrosis factor-α (TNF-α), a pro-inflammatory factor [92,93,94]. In fact, VEGF has a broad spectrum of wound healing activities, such as increased cell migration, collagen deposition, capillary growth, and epithelialization [93]. An increase of pro-inflammatory markers such as interleukin (IL)-1β, IL-6, and TNF-α leads to an upregulation of matrix metalloproteinases (MMP), particularly increased in chronic wounds [95]. MMPs degrade local extracellular matrix (ECM), impairing cell migration in wounds [96]. In resveratrol-treated patients TNF-α, IL-1β, IL-6, MMP-2, -3, -9, and C-reactive protein (CRP) were reduced [97,98,99,100].

Moreover, resveratrol has also a significant impact on the regulation of inflammation and consequently in processes related to skin repair [101]. In vitro investigations showed that due to its antioxidant properties resveratrol can influence cell proliferation, ultrastructural preservation, and migration quality [102,103], whereas in vivo studies reported a significant improvement in wound healing properties [104].

### 5.3. Resveratrol as Cardioprotective Agent

Resveratrol is a natural compound with anti-inflammatory effects. Xiuyue Huo et al. evaluated the cardio protective effects of RES in a diabetic rat model with coronary heart disease [105]. RES maintained pancreatic tissue, reducing levels of glucose and triglyceride glycerides in serum. Inflammatory factors were also suppressed by RES. TLR4/MyD88/NF-kB signalling pathway was downregulated after RES treatment [105]. To confirm the cardioprotective effects of RES, the authors harvested heat tissues from rats and examined pathological changes using H&E staining. They demonstrated that RES can offer protective effects on cardiovascular tissues in diabetic rats. By downregulating a wide range of inflammatory factors, RES strongly improved coronary injuries from inflammation. Based on this effect, RES might show a beneficial outcome or has a promising effect in diabetic cases with coronary heart disease according to its protective effects on cardiovascular tissues [105].

Resveratrol and its analogues have been shown in preclinical studies to protect against cancer treatment-induced cardiovascular toxicity. They have also been reported to possess significant anticancer properties on their own and to enhance the anticancer effect of other cancer treatments. Thus, they hold significant promise to protect the cardiovascular system and fight the cancer at the same time [106]. Thus, they hold significant promise to protect the cardiovascular system and fight the cancer at the same time. Despite the very promising preclinical findings of resveratrol as a cardioprotective agent, there are still several questions that need to be answered before advancing resveratrol into clinical trials. Considering the possible hormetic dose–response properties of resveratrol [107], it is important to show that the same dose of resveratrol can protect the heart and fight cancer. On the other hand, to determine the effect of resveratrol on the pharmacokinetics and tissue distribution of chemotherapeutic agents, considering the potential of resveratrol to alter several drug metabolizing enzymes is also rather important [108].

### 5.4. Resveratrol in Fighting Infections

Resveratrol has been reported to show anti-inflammatory, cardioprotective, and antiproliferative properties and also to affect many viruses like retroviruses, influenza A virus, and polyomavirus by altering cellular pathways influencing viral replication itself. Epstein–Barr Virus (EBV), the agent causing mononucleosis, relates to different proliferative diseases and establishes a latent and/or a lytic infection. De Leo and co-workers [109] examined the antiviral activity of RES against the EBV replicative cycle and studied the molecular targets involved. In a cellular context that allows in vitro EBV activation and lytic cycle progression, they found that RES inhibited EBV lytic genes expression and the production of viral particles in a dose-dependent manner. They demonstrated that RES decreased reactive oxygen species (ROS) levels, inhibited protein synthesis, and suppressed the EBV-induced activation of the redox-sensitive transcription factors NF–kB and AP-1 [109]. RES action causes downregulation of EBV-lytic genes at the post-transcriptional level by influencing multiple cellular targets. The molecular mechanisms involved could be divided in three parts: (1) inhibition of protein synthesis, (2) decrement of ROS levels, and (3) fast suppression of NF–kB and AP-1 activities, augmented by EBV-lytic cycle reactivation [109]. With this work, De Leo and co-authors constructed the basis for evaluating the antiviral activity of RES against EBV infection, envisaging the possibility to extend it to other types of infections.

In a recent article, Palomera-Ávalos et al. [110] demonstrated that RES induced higher expression in cytokines with regard to LPS. Oxidative stress (OS) markers indicated nonsignificant changes after LPS or RES, although for the RES-treated groups a slight increment in several study parameters was observed, reaching significance for NF-kB protein levels and iNOS expression. The authors focused on the role of long-term resveratrol treatment as a tool to increase brain defenses in ageing animals against acute LPS proinflammatory stimuli. Their results showed an improvement in cellular response against LPS injury cellular modulation response in the ageing brain. Finally, they concluded that resveratrol treatment induced a different cellular response in ageing animals when they encountered acute inflammatory stimuli [110].

Shevelev and co-workers have compared the antimicrobial activity of resveratrol (a stilbene), dihydroquercetin, and dihydromyricetin (two flavonols) extracted from the bark and wood of conifers against the dermatophytes *Staphylococcus aureus*, *Pseudomonas aeruginosa*, and *Candida albicans*. This study suggests a significantly higher bactericidal activity of polyphenols in comparison with those traditionally used against *S. aureus* at 24.2 mM [111].

It has been shown that RES can block the critical pathways involved in SARS-CoV-2 pathogenesis, including control of both RAS and ACE2, and body immune activation, and downregulate pro-inflammatory cytokine release. It was also discovered to activate SIRT1 and p53 signalling pathways and increase cytotoxic T lymphocytes (CTLs) and natural killer (NK) immune cells. RES has also been a fetal hemoglobin stimulator and a potent antioxidant capable of blocking ROS [112,113].

**Figure 7:**
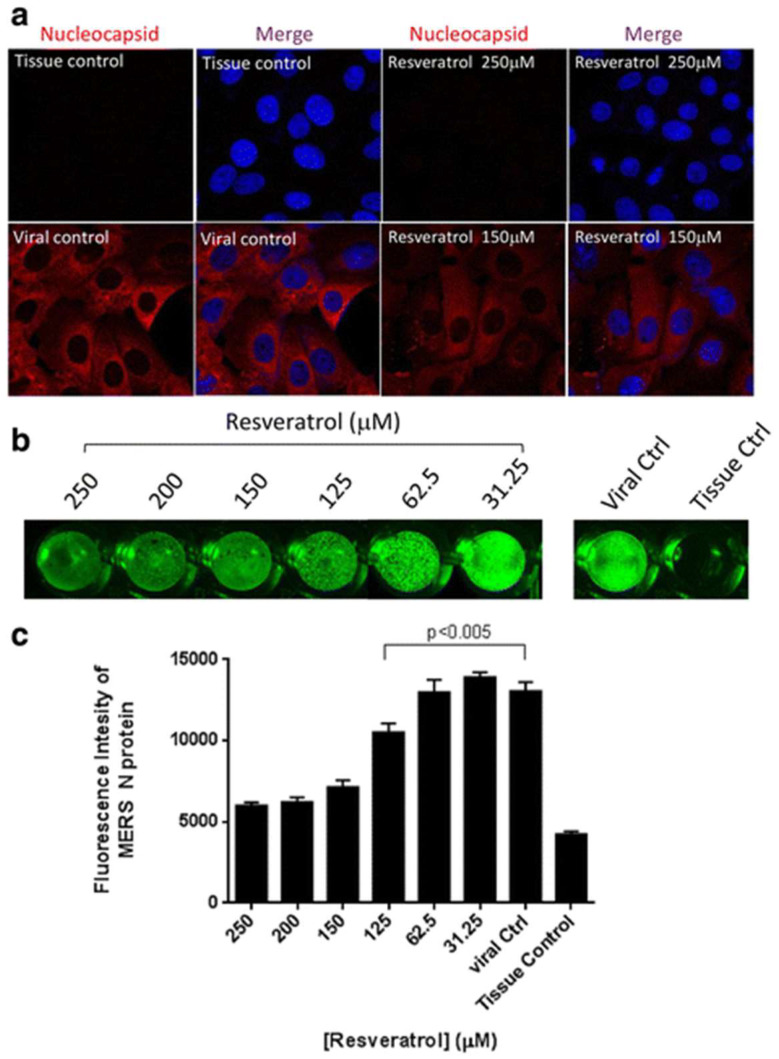
Resveratrol reduced nucleocapsid expression of MERS-CoV. Vero E6 cells were infected by MERS-CoV (M.O.I. 0.1) and treated with resveratrol for 24 h followed by 4% paraformaldehyde fixation for immunofluorescent assays. (**a**) Nucleocapsid expressions were examined with confocal microscope at 680× magnification. DAPI was used for nucleus staining. (**b**) Intracellular staining of MERS nucleocapsid expressions were visualized by Odyssey^®^ CLx Imaging system. (**c**) Quantification results of fluorescent intensities of MERS nucleocapsid proteins were determined by Odyssey^®^ CLx Imaging software. (Reproduced with permission from Ref. [115]).

RES also blocked MERS-CoV replication in vitro by inhibiting RNA synthesis and had other pleiotropic consequences, and it inhibited viral replication and reduced the death risk in piglets infected with the duck enteritis virus [114]. Similarly, Lin et al. (2017) showed that resveratrol can prevent MERS-CoV infections in a Vero E6 cell model. Two resveratrol concentrations of 250 and 125 μM were investigated, demonstrating that resveratrol can reduce the cell death induced by infection at this concentration range [115] (see Figure 7). Currently there is no evidence that RES has been used to treat SARS-CoV-2; however, available studies may envisage that it could be a strategic adjunctive antiviral agent to use in synergy with others.

## 6. Conclusions

Resveratrol is a very promising natural molecule because of its enormous therapeutic potential. However, the potential of resveratrol has not been fully explored due to its poor bioavailability. In the past two decades, nano-based delivery systems have created new hopes for delivering numerous drug molecules. Regardless of the encouraging results in preclinical trials, the suitability of resveratrol for humans has encountered only inadequate accomplishment, mostly due to its incompetent systemic delivery and, subsequently, its low bioavailability enterohepatic recirculation. Whether or not enterohepatic recirculation of resveratrol contributes expressively to the complete pharmacological action needs to be determined. To overcome these issues, it has been shown that encapsulating resveratrol into nanocarriers drastically improves the obstacles to its physicochemical characteristics. Furthermore, nanocarriers can enhance the bioavailability and permeability of resveratrol molecules, which are otherwise challenging to deliver orally.

## 7. Current Limitations and Future Perspectives

Several resveratrol-based nanoformulations are being explored for clinical applications. Despite these achievements, the ‘ideal’ resveratrol delivery system still needs to be disclosed. Significant progress has been made over last few years for developing the next generations of resveratrol-based nanocarriers that will play an essential role in curing human diseases and improving healthcare. The main limitations of these nanocarriers rely on difficulties in increasing bioavailability and targeting. By developing and designing novel nanovectors, one may also take into account the obstacles related to overcoming biological barriers in delivering cargos as well as the impendence to maintain intact or still-active, large quantities of RES during the transport to the target. For these reasons, additional studies (in vitro and more in vivo) are needed, and they will allow the development of novel strategies to overcome such challenges still limiting the use of resveratrol for therapy and/or prevention in deadly diseases such as cancer.

## Figures and Tables

**Table 1:** Brief overview of most important type of nanocarriers used for delivering resveratrol.

Nano System	Effect	Key Properties	Reference
Liposomes	Antioxidant activity	Enhanced solubility and stability of curcumin and resveratrol. Minimum particle size, lower polydispersity index and high encapsulation efficiency	[62]
	Anticancer activity	HepG2 cells exhibited a higher uptake of encapsulated-RES than the free form	[63]
Dendrimers	Enhanced RES solubility and stability in aqueous solution. Dendrimers can be engineered to control pharmacokinetics and target for oral, mucosal, transdermal, or parenteral administration	(PAMAM) dendrimer assembly overcame the problems of low bioavailability and poor water solubility	[64]
Solid lipid nanoparticles	In vitro cytotoxicity against C6 glioma cell lines	The resveratrol-TPGS-SLNs showed 11.12 and 9.37-times higher area under the curve (AUC) and plasma half-life, respectively, than the unprocessed resveratrol. Additionally, the concentration of resveratrol-TPGS-SLNs in the brain was found to be 9.23-times higher compared to free resveratrol	[65]
Polymeric nanoparticles	Fatty liver disorder	The prepared poly(lactic-*co*-glycolic acid) (PLGA) nanoparticles containing resveratrol increased its stability and solubility, yielding better in vitro results as compared to free drug	[66]

## Data Availability

Not applicable.

## References

[1] Siegel R.L., Miller K.D., Jemal A. (2017). Cancer Statistics. CA Cancer J. Clin..

[2] Okimoto R.A., Bivona T.G. (2014). Recent advances in personalized lung cancer medicine. Pers. Med..

[3] Krepler C., Xiao M., Sproesser K., Brafford P.A., Shannan B., Beqiri M., Liu Q., Xu W., Garman B., Nathanson K.L. (2016). Personalized Preclinical Trials in BRAF Inhibitor-Resistant Patient-Derived Xenograft Models Identify Second-Line Combination Therapies. Clin. Cancer Res..

[4] Hong W.K., Sporn M.B. (1997). Recent advances in chemoprevention of Cancer. Science.

[5] Sethi G., Shanmugam M.K., Ramachandran L., Kumar A.P., Tergaonkar V. (2012). Multifaceted link between Cancer and inflammation. Biosci. Rep..

[6] Chai E.Z., Siveen K.S., Shanmugam M.K., Arfuso F., Sethi G. (2015). Analysis of the intricate relationship between chronic inflammation and Cancer. Biochem. J..

[7] Sethi G., Tergaonkar V. (2009). Potential pharmacological control of the NF-κB pathway. Trends Pharmacol. Sci..

[8] Janakiram N.B., Mohammed A., Madka V., Kumar G., Rao C.V. (2016). Prevention and treatment of cancers by immune modulating nutrients. Mol. Nutr. Food Res..

[9] Sharma S., Stutzman J.D., Kelloff G.J., Steele V.E. (1994). Screening of potential chemopreventive agents using biochemical markers of carcinogenesis. Cancer Res..

[10] Martinez J., Moreno J.J. (2000). Effect of resveratrol, a natural polyphenolic compound, on reactive oxygen species and prostaglandin production. Biochem. Pharmacol..

[11] Leonard S.S., Xia C., Jiang B.H., Stinefelt B., Klandorf H., Harris G.K., Shi X. (2003). Resveratrol scavenges reactive oxygen species and effects radical-induced cellular responses. Biochem. Biophys. Res. Commun..

[12] Kim H.J., Chang E.J., Bae S.J., Shim S.M., Park H.D., Rhee C.H., Park J.H., Choi S.W. (2002). Cytotoxic and antimutagenic stilbenes from seeds of *Paeonia lactiflora*. Arch. Pharm. Res..

[13] Sgambato A., Ardito R., Faraglia B., Boninsegna A., Wolf F.I., Cittadini A. (2001). Resveratrol, a natural phenolic compound, inhibits cell proliferation and prevents oxidative DNA damage. Mutat. Res..

[14] Attia S.M. (2012). Influence of resveratrol on oxidative damage in genomic DNA and apoptosis induced by cisplatin. Mutat. Res..

[15] Ko J.H., Sethi G., Um J.Y., Shanmugam M.K., Arfuso F., Kumar A.P., Bishayee A., Ahn K.S. (2017). The role of resveratrol in cancer therapy. Int. J. Mol. Sci..

[16] Singla R.K., Dubey A.K., Garg A., Sharma R.K., Fiorino M., Ameen S.M., Haddad M.A., Al-Hiary M. (2019). Natural polyphenols: Chemical classification, definition of classes, subcategories, and structures. J. AOAC Int..

[17] Wink M. (2018). Plant secondary metabolites modulate insect behavior-steps toward addiction?. Front. Physiol..

[18] Laganà P., Anastasi G., Marano F., Piccione S., Singla R.K., Dubey A.K., Delia S., Coniglio M.A., Facciolà A., Di Pietro A. (2019). Phenolic Substances in Foods: Health Effects as Anti-Inflammatory and Antimicrobial Agents. J. AOAC Int..

[19] Wang T.Y., Li Q., Bi K.S. (2018). Bioactive flavonoids in medicinal plants: Structure, activity and biological fate. Asian J. Pharma. Sci..

[20] Evans M., Sharma P., Guthrie N. (2012). Bioavailability of citrus polymethoxylated flavones and their biological role in metabolic syndrome and hyperlipidemia. Readings in Advanced Pharmacokinetics-Theory, Methods and Applications.

[21] Testai L., Calderone V. (2017). Nutraceutical value of citrus flavanones and their implications in cardiovascular disease. Nutrients.

[22] Yu J., Bi X., Yu B., Chen D. (2016). Isoflavones: Anti-inflammatory benefit and possible caveats. Nutrients.

[23] Khoo H.E., Azlan A., Tang S.T., Lim S.M. (2017). Anthocyanidins and anthocyanins: Colored pigments as food, pharmaceutical ingredients, and the potential health benefits. Food Nutr. Res..

[24] Saibabu V., Fatima Z., Khan L.A., Hameed S. (2015). Therapeutic potential of dietary phenolic acids. Adv. Pharmacol. Sci..

[25] Rodríguez-García C., Sánchez-Quesada C., Toledo E., Delgado-Rodríguez M., Gaforio J.J. (2019). Naturally lignan-rich foods: A dietary tool for health promotion?. Molecules.

[26] El Khawand T., Courtois A., Valls J., Richard T., Krisa S. (2018). A review of dietary stilbenes: Sources and bioavailability. Phytochem. Rev..

[27] Salehi B., Mishra A.P., Nigam M., Sener B., Kilic M., Sharifi-Rad M., Fokou P.V.T., Martins N., Sharifi-Rad J. (2018). Resveratrol: A double-edged sword in health benefits. Biomedicines.

[28] Trela B.C., Waterhouse A.L. (1996). Resveratrol: Isomeric molar absorptivities and stability. J. Agric. Food Chem..

[29] Gokce E.H., Korkmaz E., Dellera E., Sandri G., Bonferoni M.C., Ozer O. (2012). Resveratrol-loaded solid lipid nanoparticles versus nanostructured lipid carriers: Evaluation of antioxidant potential for dermal applications. Int. J. Nanomed..

[30] Chen J., Wei N., Lopez-Garcia M., Ambrose D., Lee J., Annelin C., Peterson T. (2017). Development and evaluation of resveratrol, Vitamin E, and epigallocatechin gallate loaded lipid nanoparticles for skin care applications. Eur. J. Pharm. Biopharm..

[31] Nawaz W., Zhou Z., Deng S., Ma X., Ma X., Li C., Shu X. (2017). Therapeutic versatility of resveratrol derivatives. Nutrients.

[32] Li M., Kildegaard K.R., Chen Y., Rodriguez A., Borodina I., Nielsen J. (2015). De novo production of resveratrol from glucose or ethanol by engineered Saccharomyces cerevisiae. Metab. Eng..

[33] Burns J., Yokota T., Ashihara H., Lean M.E., Crozier A. (2002). Plant foods and herbal sources of resveratrol. J. Agric. Food Chem..

[34] Risuleo G. (2016). Resveratrol: Multiple activities on the biological functionality of the cell. Nutraceuticals.

[35] Neubert R.H.H. (2011). Potentials of new nanocarriers for dermal and transdermal drug delivery. Eur. J. Pharm. Biopharm..

[36] How C.W., Rasedee A., Manickam S., Rosli R. (2013). Tamoxifen-loaded nanostructured lipid carrier as a drug delivery system: Characterization, stability assessment and cytotoxicity. Colloids Surf. B. Biointerfaces.

[37] Wong H.L., Bendayan R., Rauth A.M., Li Y., Wu X.Y. (2007). Chemotherapy with anticancer drugs encapsulated in solid lipid nanoparticles. Adv. Drug Deliv. Rev..

[38] Wang A.Z., Langer R., Farokhzad O.C. (2012). Nanoparticle delivery of cancer drugs. Annu. Rev. Med..

[39] Zeb A., Qureshi O.S., Kim H.S., Kim M.S., Kang J.H., Park J.S., Kim J.K. (2017). High payload itraconazole-incorporated lipid nanoparticles with modulated release property for oral and parenteral administration. J. Pharm. Pharmacol..

[40] Kakkar D., Dumoga S., Kumar R., Chuttani K., Mishra A.K. (2015). PEGylated solid lipid nanoparticles: Design, methotrexate loading and biological evaluation in animal models. Med. Chem. Comm..

[41] Qureshi O.S., Kim H.S., Zeb A., Choi J.S., Kim H.S., Kwon J.E., Kim M.S., Kang J.H., Ryou C., Park J.S. (2017). Sustained release docetaxel-incorporated lipid nanoparticles with improved pharmacokinetics for oral and parenteral administration. J. Microencapsul..

[42] Deshpande P.P., Biswas S., Torchilin V.P. (2013). Current trends in the use of liposomes for tumor targeting. Nanomedicine.

[43] Basu S., Sandanaraj B.S., Thayumanavan S. (2002). Molecular recognition in dendrimers. Encyclopedia of Polymer Science and Technology.

[44] Lai P.S., Lou P.J., Peng C.L., Pai C.L., Yen W.N., Huang M.Y., Young T.H., Shieh M.J. (2007). Doxorubicin delivery by polyamidoamine dendrimer conjugation and photochemical internalization for cancer therapy. J. Control. Release..

[45] Zielińska A., Carreiró F., Oliveira A.M., Neves A., Pires B., Venkatesh D.N., Durazzo A., Lucarini M., Eder P., Silva A.M. (2020). Polymeric nanoparticles: Production, characterization, toxicology and ecotoxicology. Molecules.

[46] ud Din F., Aman W., Ullah I., Qureshi O.S., Mustapha O., Shafique S., Zeb A. (2017). Effective use of nanocarriers as drug delivery systems for the treatment of selected tumors. Int. J. Nanomed..

[47] Wang W., Chen S., Zhang L., Wu X., Wang J., Chen J.F., Le Y. (2015). Poly (lactic acid)/chitosan hybrid nanoparticles for controlled release of anticancer drug. Mater Sci. Eng. C..

[48] Anitha A., Deepa N., Chennazhi K.P., Lakshmanan V.K., Jayakumar R. (2014). Combinatorial anticancer effects of curcumin and 5-fluorouracil loaded thiolated chitosan nanoparticles towards colon cancer treatment. Biochim. Biophys. Acta.

[49] Zhu Y., Liao L. (2015). Applications of nanoparticles for anticancer drug delivery: A review. J. Nanosci. Nanotechnol..

[50] Biswas S., Kumari P., Lakhani P.M., Ghosh B. (2016). Recent advances in polymeric micelles for anticancer drug delivery. Eur. J. Pharm. Sci..

[51] Gothwal A., Khan I., Gupta U. (2016). Polymeric micelles: Recent advancements in the delivery of anticancer drugs. Pharm. Res..

[52] Ren J., Fang Z., Yao L., Dahmani F.Z., Yin L., Zhou J., Yao J. (2015). A micelle-like structure of poloxamer–methotrexate conjugates as nanocarrier for methotrexate delivery. Int. J. Pharma..

[53] Zhang W., Li M., Zhou W., Zhang X., Li F. (2020). Self-assembly, biosynthesis, functionalization and applications of virus-based nanomaterials. Synth. Biol. J..

[54] Slowing I.I., Vivero-Escoto J.L., Wu C.W., Lin V.S.Y. (2008). Mesoporous silica nanoparticles as controlled release drug delivery and gene transfection carriers. Adv. Drug Del. Rev..

[55] Rosenholm J.M., Peuhu E., Bate-Eya L.T., Eriksson J.E., Sahlgren C., Lindén M. (2010). Cancer-cell-specific induction of apoptosis using mesoporous silica nanoparticles as drug-delivery vectors. Small.

[56] Ng C.M., Loh H.S., Muthoosamy K., Sridewi N., Manickam S. (2016). Conjugation of insulin onto the sidewalls of single-walled carbon nanotubes through functionalization and diimide-activated amidation. Int. J. Nanomed..

[57] Vardharajula S., Ali S.Z., Tiwari P.M., Eroğlu E., Vig K., Dennis V.A., Singh S.R. (2012). Functionalized carbon nanotubes: Biomedical applications. Int. J. Nanomed..

[58] Rout G.K., Shin H.S., Gouda S., Sahoo S., Das G., Fraceto L.F., Patra J.K. (2018). Current advances in nanocarriers for biomedical research and their applications. Artif. Cells Nanomed. Biotechnol..

[59] Prabhakar N., Zhang J., Desai D., Casals E., Gulin-Sarfraz T., Näreoja T., Westermarck J., Rosenholm J.M. (2016). Stimuli-responsive hybrid nanocarriers developed by controllable integration of hyperbranched PEI with mesoporous silica nanoparticles for sustained intracellular siRNA delivery. Int. J. Nanomed..

[60] Desai D., Zhang J., Sandholm J., Lehtimäki J., Grönroos T., Tuomela J., Rosenholm J.M. (2017). Lipid bilayer-gated mesoporous silica nanocarriers for tumor-targeted delivery of zoledronic acid in vivo. Mol. Pharm..

[61] Han N., Zhao Q., Wan L., Wang Y., Gao Y., Wang P., Wang Z., Zhang J., Jiang T., Wang S. (2015). Hybrid lipid-capped mesoporous silica for stimuli-responsive drug release and overcoming multidrug resistance. ACS Appl. Mater. Interfaces.

[62] Huang M., Liang C., Tan C., Huang S., Ying R., Wang Y., Wang Z., Zhang Y. (2019). Liposome co-encapsulation as a strategy for the delivery of curcumin and resveratrol. Food Funct..

[63] Jagwani S., Jalalpure S., Dhamecha D., Jadhav K., Bohara R. (2020). Pharmacokinetic and pharmacodynamic evaluation of resveratrol loaded cationic liposomes for targeting hepatocellular carcinoma. ACS Biomater. Sci. Eng..

[64] Chauhan A.S. (2015). Dendrimer nanotechnology for enhanced formulation and controlled delivery of resveratrol. Ann. N. Y. Acad. Sci..

[65] Vijayakumar M.R., Kumari L., Patel K.K., Vuddanda P.R., Vajanthri K.Y., Mahto S.K., Singh S. (2016). Intravenous administration of trans-resveratrol-loaded TPGS-coated solid lipid nanoparticles for prolonged systemic circulation, passive brain targeting and improved in vitro cytotoxicity against C6 glioma cell lines. RSC Adv..

[66] Wan S., Zhang L., Quan Y., Wei K. (2018). Resveratrol-loaded PLGA nanoparticles: Enhanced stability, solubility and bioactivity of resveratrol for non-alcoholic fatty liver disease therapy. R. Soc. Open Sci..

[67] Swamy M.K., Sinniah U.R. (2016). Patchouli (*Pogostemon cablin* Benth.): Botany, agrotechnology and biotechnological aspects. Ind. Crops Prod..

[68] Mohanty S.K., Swamy M.K., Sinniah U.R., Anuradha M. (2017). *Leptadenia reticulata* (Retz.) Wight & Arn. (Jivanti): Botanical, agronomical, phytochemical, pharmacological, and biotechnological aspects. Molecules.

[69] Rodrigues T., Reker D., Schneider P., Schneider G. (2016). Counting on natural products for drug design. Nat. Chem..

[70] Siddiqui A.A., Iram F., Siddiqui S., Sahu K. (2014). Role of natural products in drug discovery process. Int. J. Drug Dev. Res..

[71] Beutler J.A. (2009). Natural products as a foundation for drug discovery. Curr. Prot. Pharmacol..

[72] Thilakarathna S.H., Rupasinghe H. (2013). Flavonoid bioavailability and attempts for bioavailability enhancement. Nutrients.

[73] Bonifácio B.V., da Silva P.B., dos Santos Ramos M.A., Negri K.M.S., Bauab T.M., Chorilli M. (2014). Nanotechnology-based drug delivery systems and herbal medicines: A review. Int. J. Nanomed..

[74] Watkins R., Wu L., Zhang C., Davis R.M., Xu B. (2015). Natural product-based nanomedicine: Recent advances and issues. Int. J. Nanomed..

[75] Perez A.T., Domenech G.H., Frankel C., Vogel C.L. (2002). Pegylated liposomal doxorubicin (Doxil®) for metastatic breast cancer: The Cancer Research Network, Inc., experience. Cancer Investig..

[76] Tsai Y.M., Chang-Liao W.L., Chien C.F., Lin L.C., Tsai T.H. (2012). Effects of polymer molecular weight on relative oral bioavailability of curcumin. Int. J. Nanomed..

[77] Nair H.B., Sung B., Yadav V.R., Kannappan R., Chaturvedi M.M., Aggarwal B.B. (2010). Delivery, of anti-inflammatory nutraceuticals by nanoparticles for the prevention and treatment of Cancer. Biochem. Pharmacol..

[78] Jang M., Cai L., Udeani G.O., Slowing K.V., Thomas C.F., Beecher C.W., Fong H.H., Farnsworth N.R., Kinghorn A.D., Mehta R.G. (1997). Cancer chemopreventive activity of resveratrol, a natural product derived from grapes. Science.

[79] Banerjee S., Bueso-Ramos C., Aggarwal B.B. (2002). Suppression of 7, 12-dimethylbenz (a) anthracene-induced mammary carcinogenesis in rats by resveratrol: Role of nuclear factor-κB, cyclooxygenase 2, and matrix metalloprotease 9. Cancer Res..

[80] Aggarwal B.B., Takada Y., Oommen O.V. (2004). From chemoprevention to chemotherapy: Common targets and common goals. Expert Opin. Investig. Drugs.

[81] Nguyen C., Savouret J.F., Widerak M., Corvol M.T., Rannou F. (2017). Resveratrol, potential therapeutic interest in joint disorders: A critical narrative review. Nutrients.

[82] Zhang J., Song X., Cao W., Lu J., Wang X., Wang G., Wang Z., Chen X. (2016). Autophagy and mitochondrial dysfunction in adjuvant-arthritis rats treatment with resveratrol. Sci. Rep..

[83] Bhattarai G., Poudel S.B., Kook S.-H., Lee J.-G. (2016). Resveratrol prevents alveolar bone loss in an experimental rat model of periodontitis. Acta Biomater..

[84] Wang Z.M., Chen Y.C., Wang D.P. (2016). Resveratrol, a natural antioxidant, protects monosodium iodoacetate- induced osteoarthritic pain in rats. Biomed. Pharmacother..

[85] Wu G., Wang L., Li H., Ke Y., Yao Y. (2016). Function of sustained released resveratrol on IL-1β-induced hBMSC MMP13 secretion inhibition and chondrogenic differentiation promotion. J. Biomater. Appl..

[86] Ikeda K., Torigoe T., Matsumoto Y., Fujita T., Sato N., Yotsuyanagi T. (2013). Resveratrol inhibits fibrogenesis and induces apoptosis in keloid fibroblasts. Wound Repair Regen..

[87] Pastore S., Lulli D., Fidanza P., Potapovich A.I., Kostyuk V., De Luca C., Mikhal’Chik E., Korkina L.G. (2012). Plant Polyphenols Regulate Chemokine Expression and Tissue Repair in Human Keratinocytes Through Interaction with Cytoplasmic and Nuclear Components of Epidermal Growth Factor Receptor System. Antioxid. Redox Signal..

[88] Wang H., Zhou H., Zou Y., Liu Q., Guo C., Gao G., Shao C., Gong Y. (2010). Resveratrol modulates angiogenesis through the GSK3β/β-catenin/TCF-dependent pathway in human endothelial cells. Biochem. Pharmacol..

[89] Zhou G., Han X., Wu Z., Shi Q., Bao X. (2019). Rosiglitazone accelerates wound healing by improving endothelial precursor cell function and angiogenesis in db/db mice. PeerJ.

[90] Gokce E.H., Tanrıverdi S.T., Eroglu I., Tsapis N., Gokce G., Tekmen I., Fattal E., Ozer O. (2017). Wound healing effects of collagen-laminin dermal matrix impregnated with resveratrol loaded hyaluronic acid-DPPC microparticles in diabetic rats. Eur. J. Pharm. Biopharm..

[91] Yurdagul A., Kleinedler J.J., McInnis M.C., Khandelwal A.R., Spence A.L., Orr A.W., Dugas T.R. (2014). Resveratrol promotes endothelial cell wound healing under laminar shear stress through an estrogen receptor-α-dependent pathway. Am. J. Physiol. Circ. Physiol..

[92] Yaman I., Derici H., Kara C., Kamer E., Diniz G., Ortac R., Sayin O. (2013). Effects of resveratrol on incisional wound healing in rats. Surg. Today.

[93] Brem H., Kodra A., Golinko M.S., Entero H., Stojadinovic O., Wang V.M., Sheahan C.M., Weinberg A.D., Woo S.L., Ehrlich H.P. (2009). Mechanism of Sustained Release of Vascular Endothelial Growth Factor in Accelerating Experimental Diabetic Healing. J. Investig. Dermatol..

[94] Çetinkalp Ş., Gökçe E.H., Şimşir I., Tanrıverdi S.T., Doğan F., Avcı Ç.B., Eroğlu I., Utku T., Gündüz C., Özer Ö. (2021). Comparative Evaluation of Clinical Efficacy and Safety of Collagen Laminin–Based Dermal Matrix Combined with Resveratrol Microparticles (Dermalix) and Standard Wound Care for Diabetic Foot Ulcers. Int. J. Low. Extrem. Wounds.

[95] Yager D.R., Zhang L.-Y., Liang H.-X., Diegelmann R.F., Cohen I.K. (1996). Wound Fluids from Human Pressure Ulcers Contain Elevated Matrix Metalloproteinase Levels and Activity Compared to Surgical Wound Fluids. J. Investig. Dermatol..

[96] Tarnuzzer R.W., Schultz G.S. (1996). Biochemical analysis of acute and chronic wound environments. Wound Repair Regen..

[97] Khodarahmian M., Amidi F., Moini A., Kashani L., Salahi E., Danaii-Mehrabad S., Nashtaei M.S., Mojtahedi M.F., Esfand-yari S., Sobhani A. (2021). A randomized exploratory trial to assess the effects of resveratrol on VEGF and TNF-α 2 expression in endometriosis women. J. Reprod. Immunol..

[98] Bo S., Ciccone G., Castiglione A., Gambino R., De Michieli F., Villois P., Durazzo M., Cavallo-Perin P., Cassader M. (2013). Anti- Inflammatory and Antioxidant Effects of Resveratrol in Healthy Smokers a Randomized, Double-Blind, Placebo-Controlled, Cross-Over Trial. Curr. Med. Chem..

[99] Tomé-Carneiro J., Gonzálvez M., Larrosa M., Yáñez-Gascón M.J., García-Almagro F.J., Ruiz-Ros J.A., Tomas-Barberan F., Conesa M.T.G., Espín J.C. (2012). Grape Resveratrol Increases Serum Adiponectin and Downregulates Inflammatory Genes in Peripheral Blood Mononuclear Cells: A Triple-Blind, Placebo-Controlled, One-Year Clinical Trial in Patients with Stable Coronary Artery Disease. Cardiovasc. Drugs Ther..

[100] Khojah H.M., Ahmed S., Abdel-Rahman M.S., Elhakeim E.H. (2018). Resveratrol as an effective adjuvant therapy in the management of rheumatoid arthritis: A clinical study. Clin. Rheumatol..

[101] Lin L.X., Wang P., Wang Y.T., Huang Y., Jiang L., Wang X.M. (2016). Aloe Vera and Vitis vinifera improve wound healing in an in vitro rat burn wound model. Mol. Med. Rep..

[102] Eroğlu I., Gökçe E.H., Tsapis N., Tanrıverdi S.T., Gökçe G., Fattal E., Özer Ö. (2015). Evaluation of Characteristics and in vitro antioxidant properties of RSV loaded hyaluronic acid-DPPC microparticles as a wound healing system. Coll. Surf. B Biointerfaces.

[103] Kaleci B., Koyuturk M. (2022). Efficacy of resveratrol in wound healing process by reducing oxiudative stress and promoting fibroblast cell proliferation and migration. Dermatol Ther..

[104] Poornima B., Korrapati P.S. (2017). Fabrication of chitosan-polycaprolactone composite nanofibrous scaffold for simultaneous delivery of ferulic acid and resveratrol. Carbohydr. Polym..

[105] Huo X., Zhang T., Meng Q., Li C., You B. (2019). Resveratrol Effects on a Diabetic Rat Model with Coronary Heart Disease. Med. Sci. Monit..

[106] Abdelgawad I.Y., Grant M.K.O., Zordoky B.N. (2019). Leveraging the Cardio-Protective and Anticancer Properties of Resveratrol in Cardio-Oncology. Nutrients.

[107] Calabrese E.J., Mattson M.P., Calabrese V. (2010). Resveratrol commonly displays hormesis: Occurrence and biomedical significance. Hum. Exp. Toxicol..

[108] Chow H.H., Garland L.L., Hsu C.H., Vining D.R., Chew W.M., Miller J.A., Perloff M., Crowell J.A., Alberts D.S. (2010). Resveratrol modulates drug- and carcinogen-metabolizing enzymes in a healthy volunteer study. Cancer Prev. Res..

[109] De Leo A., Arena G., Lacanna E., Oliviero G., Colavita F., Mattia E. (2012). Resveratrol inhibits Epstein Barr Virus lytic cycle in Burkitt’s lymphoma cells by affecting multiple molecular targets. Antivir. Res..

[110] Palomera-Ávalos V., Griñán-Ferré C., Izquierdo V., Camins A., Sanfeliu C., Canudas A.M., Pallàs M. (2018). Resveratrol modulates response against acute inflammatory stimuli in aged mouse brain. Exp. Gerontol..

[111] Shevelev A.B., Isakova E.P., Trubnikova E.V., La Porta N., Martens S., Medvedev O.A., Trubnikov D.V., Akbaev R.M., Biryukova Y.K., Zylkova M.V. (2018). A study of antimicrobial activity of polyphenols derived from wood. Bull. Russ. State Med. Univ..

[112] Shawon J., Akter Z., Hossen M.M., Akter Y., Sayeed A., Junaid M., Afrose S.S., Khan M.A. (2020). Current Landscape of Natural Products against Coronaviruses: Perspectives in COVID-19 Treatment and Anti-viral Mechanism. Curr. Pharm. Des..

[113] Gautam S., Gautam A., Chhetri S., Bhattarai U. (2020). Immunity Against COVID-19: Potential Role of Ayush Kwath. J. Ayurveda Integr. Med..

[114] Zhao X., Xu J., Song X., Jia R., Yin Z., Cheng A., Jia R., Zou Y., Li L., Yin L. (2016). Antiviral effect of resveratrol in ducklings infected with virulent duck enteritis virus. Antivir. Res..

[115] Lin S.C., Ho C.T., Chuo W.H., Li S., Wang T.T., Lin C.C. (2017). Effective inhibition of MERS-CoV infection by resveratrol. BMC Infect. Dis..

